# Carcinome neuroendocrine de la vessie: à propos de 5 cas

**DOI:** 10.11604/pamj.2017.26.92.11488

**Published:** 2017-02-24

**Authors:** Hicham El Bote, Abelilah El Alaoui, Ziouani Oussama, Hachem El Sayegh, Ali Iken, Lounis Benslimane, Yassine Nouini

**Affiliations:** 1 Service d’Urologie A, Hôpital Ibn Sina, Rabat, Maroc

**Keywords:** Neuroendocrine, cancer, vessie, traitement, pronostic, Neuroendocrine, cancer, bladder, treatment, prognosis

## Abstract

Le carcinome neuroendocrine de la vessie est une entité histologique rare, caractérisée par une dissémination métastatique rapide et un pronostic défavorable. Le but de ce travail était d'analyser les caractéristiques cliniques, histologiques, thérapeutiques et pronostiques du carcinome neuroendocrine de la vessie. Il s'agissait d'une étude rétrospective portant sur 5 patients colligés au service d’Urologie A, CHU Ibn Sina durant la période entre janvier 2008 et juin 2015. L’âge médian était de 63 ans. Quatre patients étaient de sexe masculin et une patiente de sexe féminin. Le carcinome neuroendocrine était pur dans quatre cas et impur ou associé à une composante urothéliale dans un cas. Deux cancers étaient métastatiques d’emblée. Une cystectomie a été réalisée dans un cas après chimiothérapie néoadjuvante, une chimioradiothérapie dans deux cas et une chimiothérapie palliative dans les deux autres. La durée médiane de survie était de 10 mois. Un seul patient était en vie, avec un recul de 20 mois. La prise en charge des carcinomes neuroendocrines de la vessie n’est pas standardisée, plusieurs moyens thérapeutiques ont été proposés : la chirurgie, la radiothérapie et la chimiothérapie.

## Abstract (en)

Neuroendocrine carcinoma of the bladder is a rare histological entity, characterized by the ability to metastasize quickly and associated with a poor prognosis. The purpose of this study was to analyze the clinical, histological, therapeutic and prognostic characteristics of neuroendocrine carcinoma of the bladder. We conducted a retrospective study collecting data from the medical records of 5 patients in the Department of Urology, at the Ibn Sina University Hospital during the period between January 2008 and June 2015. The average age was 63 years. Four males and one female were involved in the sudy. The neuroendocrine carcinoma was pure in four cases and impure or associated with urothelial component in one case. In two patients metastases were present at initial diagnosis. In one case, cystectomy was performed followed by neoadjuvant chemotherapy; chemoradiotherapy was performed in two cases and palliative chemotherapy in the other two cases. The median survival was 10 months. Only one patient was alive, with a follow-up period of 20 months. The management of neuroendocrine carcinoma of the bladder is not standardized, several therapeutic options have been proposed: surgery, radiation therapy and chemotherapy.

## Introduction

Le carcinome neuroendocrine de la vessie est une tumeur rare représentant 0,5 à 1% des tumeurs vésicales, il est d’évolution très péjorative avec un pronostic sévère. Il peut s’agir soit de tumeurs neuroendocrines pures, soit de tumeurs impures avec un contingent neuroendocrine émergeant au sein d’un carcinome urothélial. Il n’existe pas actuellement de consensus thérapeutique. Nous rapportons une série rétrospective de cinq cas de carcinome neuroendocrine et nous en détaillons à travers une étude de la littérature les aspects anatomocliniques et thérapeutiques.

## Méthodes

Nous avons étudié rétrospectivement les dossiers de cinq patients pris en charge dans le service d’urologie A du centre hospitalier universitaire Ibn Sina de Rabat entre janvier 2008 et juin 2015 pour un carcinome neuroendocrine de la vessie. Les critères pris en considération étaient l’âge au diagnostic, les signes cliniques, le stade, la stratégie thérapeutique et le suivi.

## Résultats

**Figure 1:**
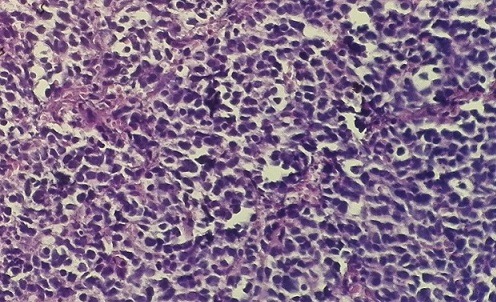
Examen anatomopathologique: coloration hématoxyline-eosine: prolifération tumorale de petites cellules basophiles

**Figure 2:**
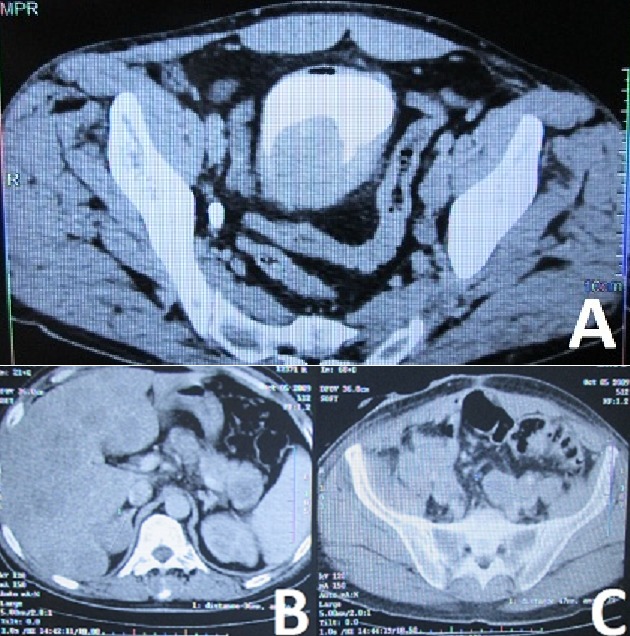
Tomodensitométrie: A) tumeur vésicale postéro latéral gauche avec infiltration de la graisse péri vésicale; B) localisations secondaires hépatiques diffuses; C) adénopathies iliaques bilatérales réalisant deux masses ilio-obturatrices engainant le sigmoïde

L’âge moyen de nos patients était de 63 ans (53 à 74 ans), quatre étaient de sexe masculin et tabagiques et une de sexe féminin non tabagique. L’hématurie était le signe cardinal dans tous les cas. Des troubles urinaires irritatifs à type de pollakiurie et dysurie ont été notés dans deux cas. La cystoscopie a montré une tumeur d’aspect solide dans la majorité des cas, localisée au niveau du trigone chez quatre patients (80%). La cystoscopie a été complétée dans tous les cas par une résection endoscopique de la tumeur. Le carcinome neuroendocrine était pur dans quatre cas et impur associé à une composante urothéliale dans un cas. L’examen anatomopathologique a objectivé une prolifération de cellules tumorales de petite taille, monomorphes, d’aspect lymphoïdes (coloration hématoxyline-éosine) (Figure 1). L’étude immunohistochimique par la chromogranine et la synaptophysine a révélé un marquage dans tous les cas. Une tomodensitométrie abdominopelvienne a été réalisée dans tous les cas (Figure 2). La scintigraphie osseuse faisait partie du bilan d’extension initial dans quatre cas. Deux cancers étaient métastatiques d’emblée, un au niveau hépatique et l’autre au niveau pulmonaire. Une cystectomie a été réalisée chez un patient suivie d’une chimiothérapie adjuvante par trois cures de cisplatine. Deux patients ont reçu une chimiothérapie par trois cures de cisplatine et d’étoposide, suivie d’une radiothérapie. Les deux autres ont reçu exclusivement une chimiothérapie. Trois cancers ont progressé sous traitement, l’évaluation n’a pu être faite pour deux patients. Seul un patient était en vie, la durée médiane de survie était de 10 mois.

## Discussion

Le poumon reste la localisation habituelle des carcinomes neuroendocrines. La localisation vésicale a été décrite pour la première fois en 1981 par Cramer et al [1, 2]. Cette atteinte est la plus fréquente au niveau de l’appareil urinaire; la prostate, le rein et l’uretère sont plus rarement concernés [1, 3]. Trois hypothèses ont été formulées pour expliquer l’origine des carcinomes neuroendocrines urinaires qui demeure inconnue: une transformation maligne, soit des cellules neuroendocrines présentes dans l’urothélium normal, soit d’une cellule souche totipotente ou une métaplasie urothéliale [4]. Le carcinome neuroendocrine de la vessie survient habituellement chez les sujets de plus de 50 ans avec une prédominance masculine (sex-ratio de 3,6) [1, 3]. Le tabagisme a été rapporté dans 67% des cas. L’hématurie est le signe clinique le plus fréquent [5]. Les mêmes constatations ont été retrouvées dans notre série. Plus rarement, des troubles mictionnels irritatifs ou un syndrome paranéoplasique (syndrome de Lambert-Eaton, myasthénie, syndrome de Cushing, hypercalcémie ou hypophos-phorémie) peuvent inaugurer la maladie [4]. Aucun de nos patients n’a souffert d’un syndrome paranéoplasique. La cystoscopie objective fréquemment des lésions polyploïdes, ulcérées et nécrotiques dont la taille varie de 4 à10 cm. Ces lésions siègent sur les parois latérales (54%), la paroi postérieure (20%), le trigone (10%), le dôme (8%) ou la paroi antérieure (8%) [3]. Dans notre série, le trigone est la localisation la plus fréquente (80%).

L’examen anatomopathologique retrouve une prolifération tumorale indifférenciée de petites cellules basophiles s’organisant en cordons, en travées ou réalisant des aspects de pseudo rosettes. L’infiltration tumorale est souvent importante avec atteinte du plan musculaire ou du tissu adipeux périvésical [6]; tel était le cas chez trois de nos patients. Dans plus de 50% des cas, une composante tumorale de type carcinome urothélial est aussi retrouvée, et plus rarement de type adénocarcinome ou carcinome épidermoïde. L’immunohistochimie confirme le diagnostic, en révèlant l’expression d’au moins un marqueur neuroendocrine (synaptophysine, chromogranine A, neurone spécifique énolase)

Le stade de la maladie est défini par la classification TNM de l’American Joint Committee on Cancer (AJCC) de 2010 [7]. Par analogie au carcinome à petites cellules du poumon, certains auteurs recommandent de distinguer des tumeurs localisées et des tumeurs disséminées, et de ne pas utiliser la classification TNM [8]. Dans notre série, le diagnostic a été fait alors que la tumeur était localement évoluée ou métastatique dans quatre cas. En effet, ces tumeurs sont agressives et sont découvertes à un stade évolué dans plus de 70% des cas et métastatiques dans 28 à 50% des cas. Les métastases ont été décrites dans les ganglions, foie, os, poumons et encéphale. Dans notre série la localisation métastatique était pulmonaire et hépatique chez deux patients. Du fait de la rareté de la maladie, il n’existe pas de standard thérapeutique [3, 6].

Dans les formes localisées plusieurs perspectives sont possibles ; l’intérêt de la cystectomie seule reste controversé, Cheng et al ont rapporté une étude de 64 patients d’après laquelle il n’y avait pas de différence en termes de survie entre les patients opérés et ceux non opérés [9]. Sved et al. ont rapporté le pronostic défavorable des cancers traités par cystoprostatectomie radicale seule [10]. Le bénéfice de la chimiothérapie néoadjuvante (quatre cures alternant ifosfamide-doxorubucine avec étoposide-cisplatine ) a été démontré dans un essai phase II du M.D. Anderson Cancer Center [11] et dans autres études rétrospectives [12]. Le seul patient survivant dans notre série a été traité par cystoprostatectomie après chimiothérapie néoadjuvante. Le rôle de la chimiothérapie adjuvante n’est pas très bien établi. La Chimiothérapie à base de cisplatine, suivie d’une radiothérapie de 56 à 70 Gy pourrait être considérée comme une alternative thérapeutique de première intention, La série rétrospective la plus large a inclus 17 patients traités au Netherlands Cancer Institute. Une réponse complète a été observée chez 15 patients (88%), avec une durée de survie médiane de 32,5 mois [13].

Dans les formes métastatiques; le traitement repose sur la chimiothérapie palliative. Les protocoles à base de cisplatine et d’étoposide sont les plus utilisés [13]. D’autres protocoles de chimiothérapie utilisant le cisplatine-étoposide en alternance avec soit ifosfamide-doxorubucine, soit doxorubucine-cyclophosphamide-vincristine peuvent être employés [5]. Le pronostic de ces tumeurs reste défavorable. La durée médiane de survie est de moins de 9 mois [3, 6]. Le taux de survie globale à cinq ans tous stades confondus est de 19% (16 à 25%). Le pronostic des carcinomes neuroendocrines purs semble être plus défavorable que celui des formes mixtes (9,5 contre 34 mois) [3, 6, 14].

## Conclusion

Les carcinomes neuroendocrines de la vessie sont des tumeurs rares, de mauvais pronostic. Le diagnostic repose sur l’examen histologique couplé à l’étude immunohistochimique. Les modalités thérapeutiques ne sont pas encore bien établies d’où la nécessite d’une concertation pluridisciplinaire.

### Etat des connaissances actuelles sur le sujet

- Le carcinome neuroendocrine de la vessie est une entité histologique rare, caractérisée par une dissémination métastatique rapide et un pronostic défavorable;
- Les modalités thérapeutiques ne sont pas encore bien établies d’où la nécessite d’une concertation pluridisciplinaire.

### Contribution de notre étude à la connaissance

- Une analyse des caractéristiques cliniques, histologiques, thérapeutiques et pronostiques du carcinome neuroendocrine de la vessie;
- Le bénéfice en matière de survie du traitement chirurgical associé à une chimiothérapie néoadjuvante versus les autres modalités thérapeutiques.
